# Imported cases of Chikungunya virus in Iran

**DOI:** 10.1186/s12879-019-4637-4

**Published:** 2019-11-27

**Authors:** Mohammad Hassan Pouriayevali, Farshid Rezaei, Tahmineh Jalali, Vahid Baniasadi, Mehdi Fazlalipour, Ehsan Mostafavi, Sahar Khakifirouz, Tahereh Mohammadi, Zahra Fereydooni, Mahsa Tavakoli, Sanam Azad-Manjiri, Motahareh Hosseini, Mahsa Ghalejoogh, Mohammad Mehdi Gouya, Anna-Bella Failloux, Mostafa Salehi-Vaziri

**Affiliations:** 1 Department of Arboviruses and Viral Hemorrhagic Fevers (National Ref Lab), Pasteur Institute of Iran, Tehran, Iran; 2 Centre for Diseases Control and Prevention, Ministry of Health, Tehran, Iran; 3 Department of Epidemiology and Biostatistics, Research Centre for Emerging and Reemerging Infectious Diseases, Pasteur Institute of Iran, Tehran, Iran; 4 Department of Virology, Institut Pasteur, Arboviruses and Insect Vectors, Paris, France; 5 Research Center for Emerging and Reemerging Infectious Diseases, Pasteur Institute of Iran, Tehran, Iran

**Keywords:** Chikungunya, Imported case, Iran, Pakistan

## Abstract

**Background:**

Chikungunya virus (CHIKV) is a widespread mosquito-borne virus representing a serious challenge to public health. The largest outbreak in the Middle-East was recorded in 2016–2017 in Pakistan. Sistan and Baluchistan Province of Iran shares a wide border with Pakistan; accordingly, introduction of CHIKV from Pakistan to Iran seems to be probable. The current study is aimed at investigating CHIKV infection in Sistan and Baluchistan Province.

**Methods:**

Between April 2017 and June 2018, a total of 159 serum samples of CHIK suspected cases from 10 cities of Sistan and Baluchistan Province were tested by molecular and serological assays. Samples obtained up to 4 days after onset of illness were tested by real time PCR (*n* = 8). Samples collected 5–10 days after disease onset were subjected to ELISA, as well as real time PCR tests (*n* = 72). Samples obtained after the 10th day of disease onset were tested by only ELISA (*n* = 79). Phylogenetic analysis of real time PCR positive samples was carried out by sequencing of a 1014-bp region of Envelope 1 gene (E1 gene). Chi-square and independent t tests were used to evaluate the association between variables and CHIKV infection.

**Results:**

In total, 40 (25.1%) out of 159 samples tested positive either by real time PCR or ELISA tests.Out of 151 samples serologically analyzed, 19 (12.6%) and 28 (18.6%) cases were positive for anti-CHIKV IgM and anti-CHIKV IgG antibodies, respectively. Of 80 samples tested by real time PCR, CHIKV RNA was detected in 11 (13.7%) sera, all of them had recent travel history to Pakistan. Additionally, phylogenetic analysis of 5 samples indicated their similarity with recent isolates of Pakistan outbreak 2016–2017 belonging to Indian Ocean sub-lineage of ECSA genotype. A significant correlation between abroad travel history and CHIKV infection was observed (*P* < 0.001). The most common clinical symptoms included fever, arthralgia/arthritis, myalgia, headache, and chill.

**Conclusions:**

These results present substantial evidence of CHIKV introduction to Iran from Pakistan and emphasize the need for the enhancement of surveillance system and preventive measures.

## Background

Chikungunya (CHIK) is a worldwide expanding mosquito-borne infection. Chikungunya virus (CHIKV), an enveloped and spherical virus, belongs to *Alphavirus* genus of *Togaviridae* family first detected in 1952 among Makonde population in Tanzania [1, 2]. CHIKV genome constitutes of a single strand positive RNA with two open reading frames (ORFs). The first ORF encodes four nonstructural proteins (nsP1, nsP2, nsP3, nsP4) and the second one encodes five structural proteins (capsid [C], and envelope [E2, E3, 6 k, E1]) [3]. Chikungunya is considered a growing global health threat as its reemergence in the 2000s led to widespread epidemics affecting millions of people in more than 60 countries in Africa, the Americas, Asia, and Europe [4]. Although the disease is rarely fatal, as its name implies, it can lead to debilitating arthralgia (in the Makonde language, Chikungunya means “that which bends up”) [3].CHIKV is transmitted to humans through infected mosquito bites of *Aedes* spp., in particular *Ae. albopictus* and *Ae. aegypti*. Similar to other arboviruses such as Dengue and Zika, CHIKV can be transmitted via the urban transmission cycle between humans and mosquito [5]. In the majority of infected individuals, there is a four-to seven-day incubation period prior to onset of disease. The main symptoms of CHIKV infection are the sudden onset of high fever (102 °F) and severe arthralgia; other symptoms include headaches, muscle aches, nausea, vomiting, arthritis, and skin rash. The clinical signs of the acute form can be mild, moderate, and severe, but usually resolve within 3 weeks. About 10–15% of cases develop chronic forms associated with persistent arthralgia [4, 6].

The first record of CHIKV circulation in the Middle-East dates back to 1981 in Pakistan [7]. Since then, recurrent outbreaks in Pakistan, Saudi Arabia, and Yemen are reported in this region [8–11]. The largest outbreaks in this region leading to over 30,000 infected cases occurred in 2016–2017 in the Karachi City of Pakistan [12].

An important factor facilitating the spread of CHIKV is the increase in international travel, which consequently introduces the virus through viremic travelers to new regions [3]. If the vectors are present in such regions, they could acquire the virus, while feeding on viremic travelers. This subsequently can result in autochthonous transmission making those areas endemic for the disease. Sistan and Baluchistan Province of Iran shares a border with Pakistan and the residents of the Southern part of this province have significant cultural similarities with those of Pakistan and, hence, constant trade and transport between the two countries is very common. Consequently, virus introduction from Pakistan to Iran due to the large incidence of travel is likely. According to the report on detection of *Ae. albopictus* in Sistan and Baluchistan Province [13] and the suitability of this province to establish the mosquitoes with the potential to transmit CHIKV [14], virus importation via travelers could be a serious health threat. The current study aimed at investigating the presence of CHIKV in suspected individuals in Sistan and Baluchistan Province during the outbreak in 2017 in Pakistan.

## Methods

### Ethical statement

The current retrospective study was conducted on samples collected in the context of National Surveillance Program of Iran for Aedes-borne arboviral infections in accordance with the protocols approved by Iranian Centre for Disease Control and Management Committee. Due to the retrospective nature of the study, it was not possible to obtain informed consent from the participants; however, all data were analyzed anonymously and all experiments were carried out according to the relevant laws and guidelines of the ethical standards of the Declaration of Helsinki.

### Study area

The current cross-sectional study was conducted in Sistan and Baluchistan Province of Iran. This province, located in Southeastern Iran with an area of 180,726 km^2^, is the only province of Iran sharing border with Pakistan. The climate in this province varies from moderate in North to semi tropical in South. It has the lowest rainfall from April to November and the southern part is affected by monsoons, which cause extensive rainfall and flooding every three to 5 years [15].

### Data and sample collection

**Fig. 1:**
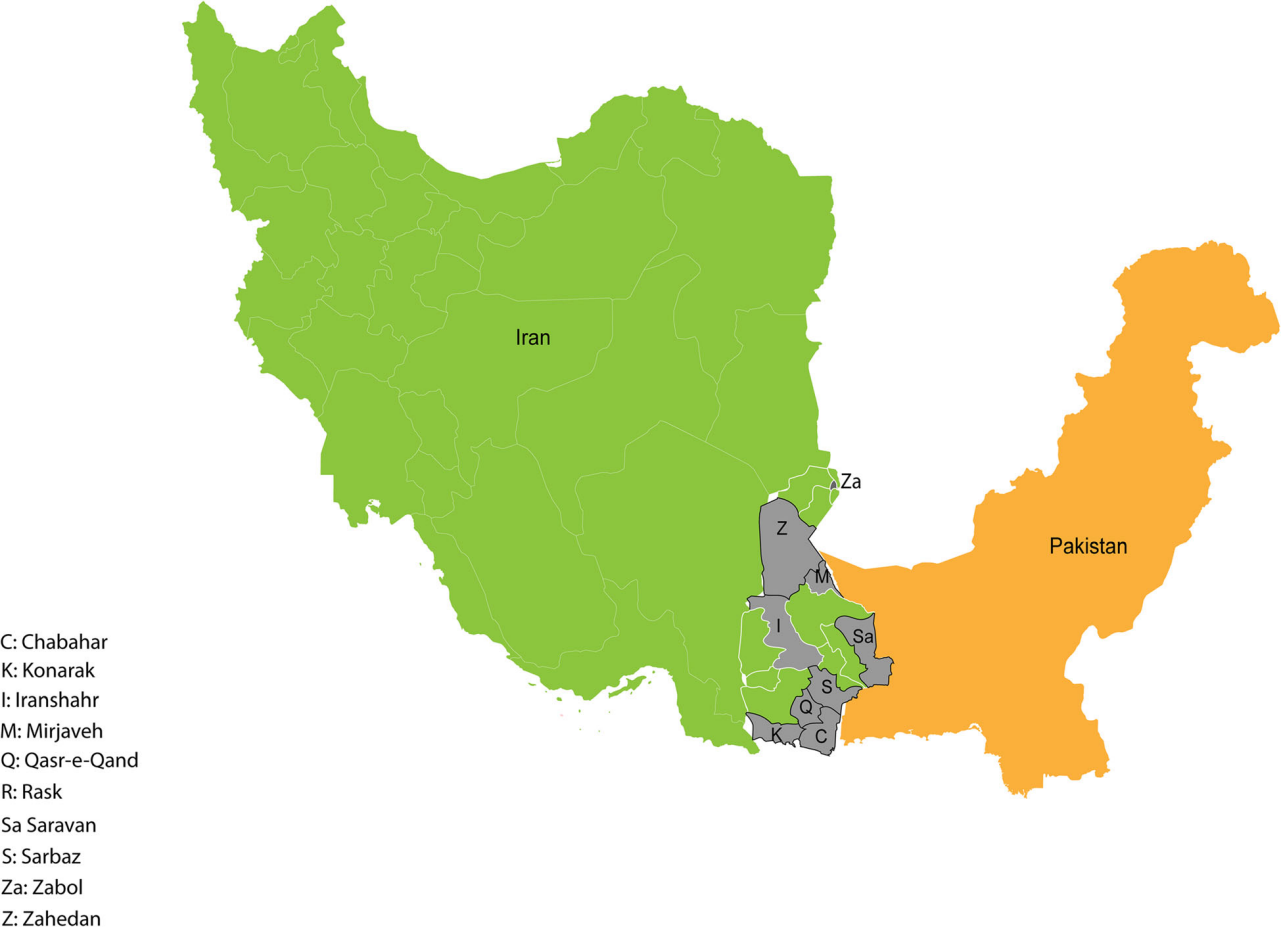
Geographical location of Sistan and Baluchistan Province and sampling areas, 2017 to 2018. This figure was originally created in this study

From April 2017 to June 2018, a total of 159 serum samples of patients suspected of CHIKV infection (febrile individuals with arthralgia or arthritis) collected within Iranian National Surveillance Program from 10 cities (Fig. 1) including Chabahar, Iranshahr, Konarak, Mirjaveh, Qasr-e-Qand, Rask, Saravan, Sarbaz, Zabol, and Zahedan were assessed for CHIKV infection. Demographic, epidemiologic, clinical, and laboratory data were collected through questionnaires and patients records.

### Laboratory diagnosis

Diagnostic algorithm was determined based on the interval between dates of sampling and onset of disease. In CHIKV infection, viremia starts before onset of symptoms and usually lasts up to 8 days after illness. Anti-CHIKV antibodies can usually be identifiable in serum by 5–7 days after onset of symptoms [16]. Therefore, samples collected up to 4 days after onset of disease were subjected to molecular test for viral RNA detection (*n* = 8). Samples obtained 5–10 days post onset of disease were tested by serological assays to detect anti-CHIKV IgM and IgG antibodies, as well as molecular assay (*n* = 72). Samples collected after the 10th day of disease onset were analyzed by only serological assays (*n* = 79). All CHIKV diagnostic assays were performed at the Department of Arboviruses and Viral Hemorrhagic Fevers (National Ref. Lab), Pasteur Institute of Iran.

For molecular detection of CHIKV, viral RNA extraction from serum samples was performed using QIAamp viral RNA Kit (Qiagen, Germany) according to the manufacturer’s instruction. The presence of the CHIKV RNA was evaluated using FTD commercial Dengue/Chik kit (Fast Track Diagnostics, Luxembourg) based on qualitative TaqMan real-time PCR. This kit contains an internal control to ensure the avoidance of false negative results. For phylogenetic analysis of positive molecular cases, a 1014-bp region of E1 gene of CHIKV genome was amplified by a conventional RT-PCR using primers Chik-E1-F-10004 (ACAAAACCGTCATCCCGTCTC) and Chik-E1-R-11138 (TGACTATGTGGTCCTTCGGAGG) and Qiagen One Step RT-PCR Kit (Qiagen, Germany) [17]. Each 25 μL of reaction mixture, contained 5 μL 9QIAGEN OneStep RT-PCR Buffer (5x), 0.4 μM of each primer, 10 μL dNTP Mix (containing 10 mM of each dNTP), 1 μL Enzyme Mix, 0.25 μL(or 1 unit) RNase inhibitor, and 2.5 μL extracted RNA. The PCR conditions were as follows: reverse transcription at 50 °C for 30 min, 15 min initial denaturation at 95 °C, 45 cycles including a) denaturation at 95 °C for 30 s, b) annealing at 50 °C for 30 s, and c) extension at 72 °C for 70 s followed by a final extension at 72 °C for 10 min. The RT-PCR products were electrophoresed on 1% agarose gel containing 0.008% SafeStain (Takapouzist, Iran). RNAs extracted from confirmed CHKV-positive sample and sterile distilled water were used as positive and negative controls, respectively. The amplified products were sequenced using the Sanger method in both directions using primers Chik-E1-F-10004 and Chik-E1-R-11138. The raw data of sequencing was trimmed and assembled using CLC Main Workbench software (CLC bio, Denmark) followed by confirmation by BLAST (https://blast.ncbi.nlm.nih.gov/Blast.cgi). Phylogenetic analysis was performed using MEGA6. For this purpose, the maximum likelihood methodand the kimura 2-parameter model were used with 10,000 bootstraps.

Detection of anti-CHIKV IgM/IgG antibodies was performed using commercial ELISA (enzyme-linked immunosorbent assay) kit (Euroimmun, Germany) according to the manufacturer’s instructions. Based on the protocol, results were reported as a ratio of the optical density of each sample to the optical density of the calibrator in which a ratio of ≤0.8 was considered negative, a ratio between ≥0.8 and ≤ 1.1 was considered borderline, and a ratio of > 1.1 was interpreted as positive.

### Statistical analysis

Statistical analysis was performed using SPSS software (version 19). The results of the serological and molecular tests were analyzed positively and negatively and the borderline results were considered as negative. Chi-square and independent ttests were used to check the relationship between variables and CHIKV infection. *P-*values less than 0.05 were considered statistically significant.

## Results

According to diagnosis algorithm, eight samples were tested only by molecular assay (category 1), 79 samples were analyzed by only serological assay (category 2), and 72 samples were investigated by both molecular and serological assays (category 3).

Of the 151 samples (category 2, 3) serologically analyzed for anti-CHIKV IgM/G antibodies, 12 (7.9%) were borderline, and 19 (12.6%) were positive for anti- CHIKV IgM; and 28 (18.6%) cases were positive for anti-CHIKV IgG.

**Fig. 2:**
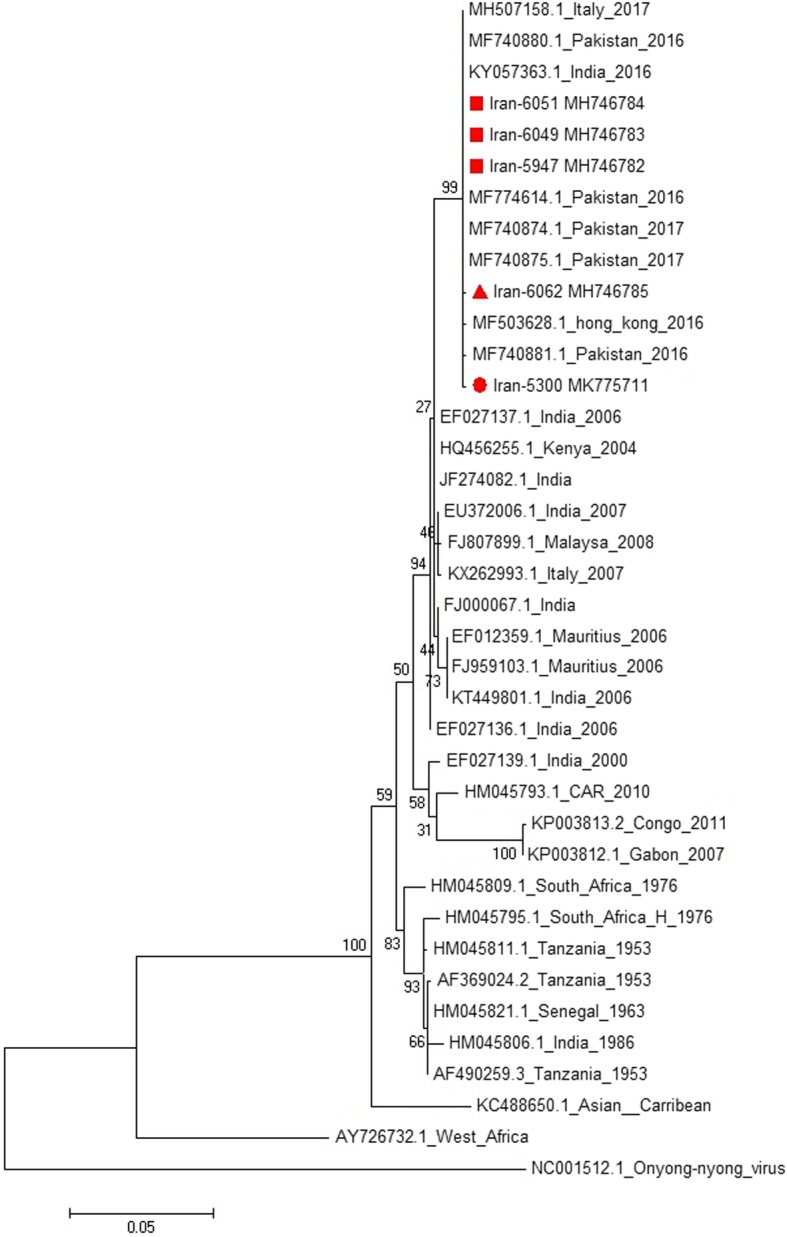
Phylogenetic tree showing high similarity between strains detected in this study (Iran-6051, Iran 6049, Iran-5947, Iran-6062 and Iran-5300) and strains identified during the Pakistan outbreak of 2016–2017

Molecular analysis of 80 (category 1, 3) serum samples showed presence of the CHIKV RNA in 11 (13.7%) sera; all of them were negative for dengue virus. Among CHIKV RNA positive samples, five were successfully sequenced. The obtained sequences were submitted to the GenBank as Iran-5947 (gi: MH746782), Iran-6049 (gi: MH746783), Iran-6051 (gi: MH746784), Iran-6062 (gi: MH746785), and Iran-5300 (gi: MK775711). In the phylogenic tree, these sequences clustered with recent isolates of Pakistan outbreak 2016–2017 belonging to Indian Ocean sub-lineage of ECSA (East/Central/South African) genotype (Fig. 2). The strains Iran-5947, Iran-6049, and Iran-6051 were identical to strains of the recent outbreak in Pakistan (MF740875- MF740881) [18]. Iran-6062 strain showed one new amino acid substitution of I/T nonpolar hydrophobic amino acid, isoleucine, to polar amino acid, threonine, at position 3593 amino acid residue (10,847 nt, ATT/ACT) compared with Pakistan 206–2017 strains [18]. Also, Iran-5300 strain showed a rare non-synonymous substitution T/C at nucleotide 10,560, which was observed in Asian Caribbean strain (KC488650). In addition, four known mutations including K211E, M269 V, D284E, and V322A were observed in all identified strains.

These data showed high similarity between strains detected in the current study (Iran-6051, Iran 6049, Iran-5947, Iran-6062 and Iran-5300) and strains identified during the Pakistan outbreak of 2016–2017.

Overall, from 159 samples assessed for CHIKV infection (by both serological and molecular assays), 40 cases (25.1%) were positive. The majority of positive cases (65%) were residents of Sarbaz. As shown in Table 1, there was a significant correlation between abroad travel history and CHIKV infection (*P* < 0.001). All CHIKV positive cases had a history of traveling abroad and molecularly-tested positive cases had a recent travel history (less than 2 weeks prior to the date of sample collection) to Pakistan. In addition to fever and arthralgia/arthritis, myalgia, headache, and chill were the most common clinical symptoms. Most of infected cases were identified in spring (47.5%) and summer (20%) (*P* = 0.042).

**Table 1:** Association of CHIKV infection with characteristics of participants, from Sistan and Baluchistan province, southeastern Iran, 2017–2018

Variables	Chik. Neg. (*n* = 119) (%)	Chik. Pos. (*n* = 40) (%)	*p*-value
Gender			
Female	57 (73.1)	21 (26.9)	0.584
Male	62 (76.5)	19 (23.5)	
Age (Mean ± SD)	31.7 ± 15.3	41.5 ± 16.3	0.001
Epidemiological History			
Abroad travelling history, N (%)	21 (17.6)	40 (100)	< 0.001
Travel Duration (day) (Mean ± SD)	15.7 ± 16.7	26.83 ± 39.3	0.218
Country with a travel history			
Afghanistan	2 (1.7)	0 (0)	0.230
Malaysia	1 (0.8)	0 (0)	0.426
Pakistan	18 (15)	40 (100)	0.001
City of residence			
Chabahar	7 (5.9)	4 (10)	0.367
Iranshahr	14 (11.7)	2 (5)	0.224
Konarak	3 (2.5)	0 (0)	0.313
Mirjaveh	2 (1.7)	0 (0)	0.411
Qasr-e Qand	1 (0.8)	0 (0)	0.562
Rask	1 (0.8)	0 (0)	0.562
Saravan	4 (3.4)	0 (0)	0.242
Sarbaz	50 (42)	26 (65)	0.010
Zabol	1 (0.8)	0 (0)	0.562
Zahedan	36 (30.2)	8(20)	0.220
Villager	57 (47.9)	19 (47.5)	0.451
Season of Symptom Onset			
Spring	34 (28.6)	19 (47.5)	0.042
Summer	48 (40.3)	8 (20)	
Fall	17 (14.3)	0 (0)	
Winter	7 (5.9)	2 (5)	
Mosquito bite	30 (25.2)	8 (20)	0.096
Clinical signs			
Chill	1 (0.8)	7 (17.5)	< 0.001
Myalgia	40 (33.6)	20 (50)	0.321
Rash	5 (4.2)	2 (5)	0.646
Headache	34 (28.6)	11 (27.5)	0.020
Conjunctivitis	11 (9.2)	2 (5)	0.064
Retro orbital pain	14 (11.7)	3 (7.5)	0.078
Stomachache	10 (8.4)	3 (7.5)	0.426
Nausea	18 (15.1)	7 (17.5)	0.658
Vomiting	17 (14.3)	7 (17.5)	0.679
Diarrhea	12 (10)	7 (17.5)	0.608
Laboratory findings			
Leukopenia	9 (7.5)	2 (5)	0.191
Platelet count(Mean ± SD)	134,486 ± 113,962	131,730 ± 126,948	0.958
WBC count(Mean ± SD)	9940 ± 1720	4980 ± 140)	0.353

## Discussion

CHIK is considered as a global health threat due to the factors associated with its geographical expansion including global warming/climate change, globalization with significant increase in international travels, and adaptation of virus to new vectors [1, 2]. Extensive epidemics in Asia and Africa could be a potential risk for its spread to other non-endemic countries in the world, particularly the ones sharing the same climate or bordering other countries that mosquito vectors already colonized or could be colonized [19]. In the current study, out of 159 patients with fever and arthralgia/arthritis in Sistan and Baluchistan Province, 25.1% were positive for either viral genome or anti-CHIKV antibodies. Unlike molecular detection of viral genome, due to possible cross-reactivity in serological testing, the positive result in ELISA cannot be concluded as a definitive infection and should be confirmed by the supplementary neutralization test [20]. In the current study, CHIKV genome was detected in sera of 11 patients, in which all had a recent travel history to Pakistan, where a widespread epidemic of the disease was ongoing at the time of the current study [21].

The phylogenetic analysis revealed significant similarity between the identified strains in the current study to those of Pakistan 2017 strains. Consequently, there is little doubt that the virus was imported to Iran from the CHIKV endemic neighboring country of Pakistan. Phylogenetically, CHIKV is categorized into three distinct genotypes e.g., West African, ECSA, and Asian [22]. Recently, a descendant lineage of ECSA, i.e., the Indian Ocean Lineage (IOL) is also identified. This lineage was responsible for several outbreaks in Southeast Asian islands from 2005 to 2014 [23, 24]. It is suggested that evolutionary mutations in different genotypes can affect the ability of virus in vector adaptation and, hence, its transmission pattern [25]*.* In the current study, a new nonsynonymous mutation in E1 gene (T288I) was observed in the Iran-6062 (MH746785) strain. The E1 protein is responsible for fusion of the viral envelope and cellular membrane, which is part of viral entry stage [26]. Mutations in E1 protein, even in a single residue, may affect vector specificity of the virus as Tsetsarkin et al., [25] demonstrated a direct association between E1-A226V mutation and adaptation of CHIKV to *Ae. albopictus*. Accordingly, there is a potential for the mutation found here to be functionally important; however, this requires further investigations. The study also detected CHIKV strains harboring E1-K211E implicated in facilitating endosomal entry of virus in *Ae. Aegypti* in background of E1-226A [27]. Nonetheless, vector competence was not only dependent on viral mutations. It is suggested that alongside virus genotype, vector genotype and environmental factors such as temperature also play a role in virus adaptation to vectors [28, 29].

There are several reports on the importation of arboviruses such as CHIK by travelers to other countries [19]. In case the vector is present in the destination country, it is likely that the virus is transmitted to vectors and, therefore, the disease could become endemic in that country. The first CHIK outbreak in Italy is a typical example of this issue. In 2007, CHIKV was reportedly introduced to Italy by a viremic worker traveling from India to the Northeast part of the country where *Ae. albopictus* mosquitoes were already established [30].

The entomological surveillance conducted alongside the current study did not find the CHIKV vectors in the studied areas. However, during a six-year entomological surveillance in Sistan and Baluchistan Province, adult *Ae. albopictus* was detected in Nikshahr and Sarbaz cities in 2009 and adult and larvae of the same species were identified in Chabahar in 2013 [13]. This finding indicated favorable conditions for the establishment of CHIKV vector in this area. Additionally, modeling studies using geographic, metrologic, satellite imagery, and remote sensing data from 2011 to 2015 demonstrated that this province was vulnerable to entry and establishment of CHIKV vectors, particularly *Ae. Albopictus* [14, 31]. Therefore, the first and foremost step to prevent outbreaks of the disease in the country is to block vector establishment. This can be accomplished by improving the environmental conditions in order to eliminate the mosquito breeding sites, and systematic and comprehensive entomological monitoring. Educating and training people is also a great help to achieve this goal. In addition to the above measures, it is also important to monitor patients and identify infected cases in the acute phase of the disease (when the transmission of the virus from the patient to the vector is possible). In this regard, training clinicians and people is essential for rapid identification of the infected cases [32].

In addition to Pakistan, there are other possible routes of virus entry to the country. According to the Department of Arboviruses and Viral Hemorrhagic Fevers at Pasteur Institute of Iran (National Reference Laboratory), the first case of CHIK was imported from India in September 2016 (unpublished data). Moreover, numerous imported cases of Dengue are recorded returning from Southeast Asian countries. Therefore, informing tourists and travelers to endemic countries to take precautions such as using insect repellents, wearing long-sleeved cloths, and using mosquito nets can play a significant role in reducing the number of infections.

In the current study, 67.5% of positive cases were infected in the spring and summer, when there is an increase in the mosquito activity and reproduction. Most of the positive cases (65%) were from Sarbaz. This can be on the account of that the majority of participants (47.8%) were from this city.

Unlike other arboviral diseases, most of the patients with CHIK have clinical symptoms [3] and the most common symptoms include severe fever (above 102 °F), arthralgia, headache, and muscle pain [5]. These are predictive symptoms in endemic and epidemic areas [33]. The results of the current study also indicated that together with joint involvement, headache, and muscle pain were the most common symptoms. In addition to clinical symptoms, changes in blood factors such as lymphopenia and thrombocytopenia are also helpful for clinical diagnosis [34]. Nevertheless, these changes are mild and short compared to other arboviral diseases such as dengue [35, 36]. In the current study, mild leukopenia and thrombocytopenia were observed in positive cases; however, there was no significant difference between the two groups.

## Conclusions

The results of the current study provide evidence of CHIKV in Iran, which were closely related to the strains in Pakistan. This highlights the urgent need to enhance the surveillance system and implement preventive measures.

## Data Availability

The datasets used and analyzed during the current study are available from the corresponding author on reasonable request.

## Abbreviations

*Ae*: *Aedes*
CHIK: Chikungunya
CHIKV: Chikungunya virus
E1 gene: Envelope 1 gene
ECSA: East Central South African
ELISA: Enzyme-Linked Immunosorbent Assay
FTD: Fast Track Diagnostics
IOL: Indian Ocean Lineage
